# Optogenetic regulation of artificial microRNA improves H_2_ production in green alga *Chlamydomonas reinhardtii*

**DOI:** 10.1186/s13068-017-0941-7

**Published:** 2017-11-07

**Authors:** Yuting Wang, Xinqin Jiang, Changxing Hu, Ting Sun, Zhiyong Zeng, Xiaoqi Cai, Hui Li, Zhangli Hu

**Affiliations:** 1 Guangdong Technology Research Center for Marine Algal Bioengineering, Guangdong Key Laboratory of Plant Epigenetics, College of Life Sciences and Oceanography, Shenzhen University, Shenzhen, 518060 People’s Republic of China; 2 Key Laboratory of Optoelectronic Devices and Systems of Ministry of Education and Guangdong Province, College of Optoelectronic Engineering, Shenzhen University, Shenzhen, 518060 People’s Republic of China; 3 Shenzhen Key Laboratory of Marine Bioresource & Eco-environmental Sciences, Longhua Innovation Institute for Biotechnology, College of Life Sciences and Oceanography, Shenzhen University, Shenzhen, 518060 People’s Republic of China

**Keywords:** Optogenetic, Light-inducible system, MicroRNA, Bio-hydrogen production, Microalga, *Chlamydomonas reinhardtii*

## Abstract

**Background:**

*Chlamydomonas reinhardtii* is an ideal model organism not only for the study of basic metabolic processes in both plants and animals but also the production of biofuels including hydrogen. Transgenic analysis of *C. reinhardtii* is now well established and very convenient, but inducible exogenous gene expression systems remain under-studied. The most commonly used heat shock-inducible system has serious effects on algal cell growth and is difficult and costly to control in large-scale culture. Previous studies of hydrogen photoproduction in *Chlamydomonas* also use this heat-inducible system to activate target gene transcription and hydrogen synthesis.

**Results:**

Here we describe a blue light-inducible system with which we achieved optogenetic regulation of target gene expression in *C. reinhardtii*. This light-inducible system was engineered in a photosynthetic organism for the first time. The photo-inducible heterodimerizing proteins CRY2 and CIB1 were fused to VP16 transcription activation domain and the GAL4 DNA-binding domain, respectively. This scheme allows for transcription activation of the target gene downstream of the activation sequence in response to blue light. Using this system, we successfully engineered blue light-inducible hydrogen-producing transgenic alga. The transgenic alga was cultured under red light and grew approximately normally until logarithmic phase. When illuminated with blue light, the transgenic alga expressed the artificial miRNA targeting photosynthetic system D1 protein, and altered hydrogen production was observed.

**Conclusions:**

The light-inducible system successfully activated the artificial miRNA and, consequently, regulation of its target gene under blue light. Moreover, hydrogen production was enhanced using this system, indicating a more convenient and efficient approach for gene expression regulation in large-scale microalgae cultivation. This optogenetic gene control system is a useful tool for gene regulation and also establishes a novel way to improve hydrogen production in green algae.

**Electronic supplementary material:**

The online version of this article (10.1186/s13068-017-0941-7) contains supplementary material, which is available to authorized users.

## Background

Bio-hydrogen production by green algae has been known since the 1940s [1] and has many advantages; for instance, it is energy-saving and environmentally friendly. However, the industrialization of hydrogen production by green algae has progressed slowly. Because oxygen induces inhibition of hydrogenase activity, green algae produce hydrogen for only a few seconds to a few minutes. To circumvent this limitation, a two-stage method based on sulfur deprivation was developed [2]. Sulfur deprivation relieves oxygen-induced inhibition of hydrogenase by reversibly reducing the rate of oxygenic photosynthesis. In the two-stage process, green algae are cultured in sulfur-replete medium until reaching a density of 3–6 million cells ml^−1^; the algal cells are then separated from the medium and cultured in sulfur-deprived medium to activate hydrogen production [3]. Since free algal cell separation and medium replacement are costly and inconvenient in large-scale culture, hydrogen photoproduction research has focused on extending the duration of hydrogen production in green algae without replacing the medium. The mRNAs [4, 5], proteins [6], miRNAs [7, 8], and lncRNAs [9] of sulfur-deprived algal cells have been studied to monitor the changes induced by sulfur deprivation. Our group previously constructed an artificial miRNA targeting the photosystem II (PS II)-related protein OEE2 (oxygen evolving enhancer 2), which is down-regulated after sulfur deprivation. Transgenic alga expressing the artificial miRNA produced hydrogen sustainably for 2 days, and had higher hydrogen yield, because oxygen consumption occurred more rapidly [10].

Although the artificial miRNA successfully extended hydrogen production time, its expression was based on a heat-inducible system of *Chlamydomonas reinhardtii*, which affects algal cell growth. *C. reinhardtii* is a photosynthetic unicellular alga and has features inherited from the common ancestor of plants and animals that were subsequently lost in land plants. It is an ideal model organism for studying many basic metabolic processes, such as chloroplast-based photosynthesis; the structure and function of eukaryotic flagella; and biofuels production [11–13]. Although transgenic technology in *C. reinhardtii* has been thoroughly developed [14, 15], inducible exogenous gene expression systems in *C. reinhardtii* remain limited. A popular system is one that employs the heat-inducible *HSP70A*-*RBCS2* promoter, which is the system we used previously to express artificial miRNAs. It can be activated by both light and heat shock [16], but the effect of light induction is limited, and heat shock compromises algal cell growth. We therefore engineered a blue light-inducible expression system into alga in the present study.

Light-inducible systems have been established in bacteria [17], cyanobacteria [18, 19], yeast [20], and mammalian cells [21, 22] but not in plants including green algae. Compared to heat shock in large-scale green algae culture, light-inducible systems would allow for faster induction, and the control of exogenous gene expression would be more uniform and more efficient. Moreover, light would not have the same detrimental effect on algal cell growth activity as heat shock, and energy consumption would be reduced in industrialized cultivation.

We developed the blue light-inducible gene expression system based on two-hybrid protein interaction. Specifically, *Arabidopsis* CRY2 (cryptochrome 2) and CIB1 (cryptochrome-interacting basic-helix-loop-helix) proteins [23] were fused with VP16 transcription activation domain and the GAL4 DNA-binding domain (GAL4 BD), respectively. CRY2 and CIB1 dimerize in blue light and dissociate within minutes in the dark. GAL4 BD recognizes and binds a specific DNA upstream activation sequence (UAS), while VP16 activation domain activates downstream gene expression. In blue light, the CRY2/CIB1 interaction therefore brings VP16 activation domain and GAL4 BD into close proximity. The UAS was bound by GAL4 BD, and the downstream gene was activated by VP16 activation domain; in the dark, CRY2-VP16 dissociated from CIB1-GAL4 BD, and the activation of the gene was reversed [20].

In this study, we used the blue light-inducible expression system to optogenetically regulate an artificial miRNA (amiR-D1) targeting the PS II reaction-center protein D1 (encoded by *psbA*) in green microalga *C. reinhardtii*. Quantitative PCR analysis showed that the transcription level of amiR-D1 was up-regulated and its target gene *psbA* was down-regulated after blue light treatment. Moreover, an improved hydrogen yield following the blue light irradiation was observed in these transgenic algae.

## Methods

### Algal strain and culture conditions

Cell-wall-deficient *C. reinhardtii* strain CC-849 was obtained from the *Chlamydomonas* Genetic Center of Duke University (Duke University, Durham, NC, USA) and served as the receptor strain and negative control. Algal cells were cultured in TAP (Tris–acetate-phosphate) medium at 22 °C under continuous cool-white or blue light fluorescent lamps or in a custom-built LED red light incubator with continuous illumination (see Additional file 1: Figure S1 for the red light wavelength distribution). The light intensities of red, blue, and white light were adjusted to similar intensity, which was about 30 μmol photons m^−2^ s^−1^.

### Blue light-inducible system vectors construction

The expression vector pH124, which was used as the expression vector backbone, contains the heat-inducible *HSP70A*-*RBCS2* promoter and the *ble* gene, which leads to algal zeocin resistance. To express the target gene constitutively, the *HSP70A*-*RBCS2* promoter and *RBCS2* 3′ UTR were replaced with the *PsaD* 5′ and *PsaD* 3′ UTR sequences, respectively. The *ble* gene was replaced with the *aph7*″ gene to confer hygromycin B resistance. The artificial mature miRNA sequence was integrated into the backbone of cre-miRNA1162, a highly expressed natural miRNA precursor. The constructed miRNA precursor sequence was commercially synthesized in vitro and cloned in plasmid pUC57.

**Fig. 1:**
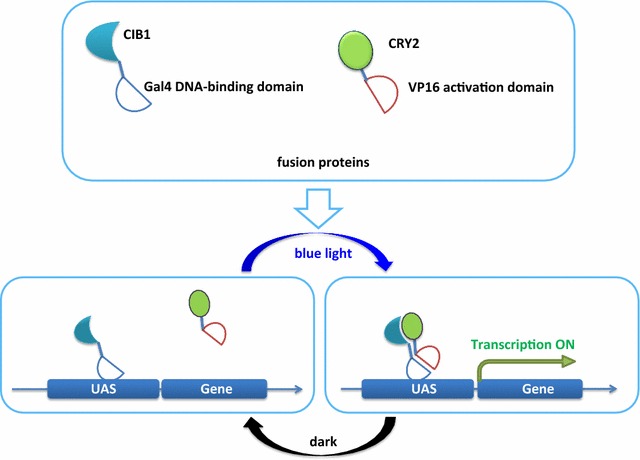
Schematic of the blue light-inducible exogenous gene expression system in *C. reinhardtii*. The GAL4 BD domain and VP16 activator are fused with CIB1 and CRY2, respectively. Blue light enables CIB1 and CRY2 heterodimerization and brings GAL4 BD and VP16 together to activate downstream gene transcription. The absence of blue light reverses the interaction. *UAS* upstream activation sequence

**Fig. 2:**
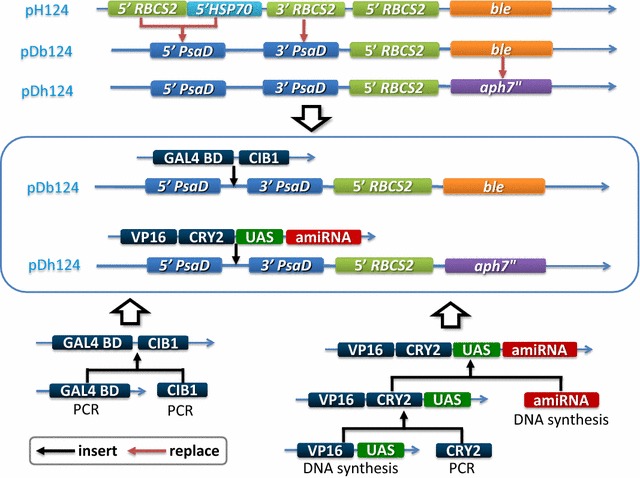
Construction of vectors in the blue light-inducible exogenous gene expression system. The top portion of the figure diagrams the promoter and resistance modification elements of the vector backbone. The bottom portion diagrams the fusion protein fragments. The middle portion shows the construction of the two vectors used in the blue light-inducible exogenous gene expression system. Red and black arrows indicate the replacement and insertion of fragments, respectively

The full-length coding sequences of *CIB1* and *CRY2* were amplified from *Arabidopsis* cDNA. The GAL4 BD was amplified from the yeast two-hybrid system vector. The miR-D1 fragment and tandem fragments of VP16 and UAS were synthesized in vitro. All of the fragments were digested and ligated step by step into the two vectors (Figs. 1, 2).

### *Chlamydomonas reinhardtii* transformation

Algal cells (2 × 10^6^ cells ml^−1^) were centrifuged and resuspended to a final concentration of 2 × 10^8^ cells ml^−1^. A 300 μl volume of algal cells was transformed with 1 μg plasmid DNA using the glass bead vortexing method. The cells were placed in 10 ml fresh TAP medium under white light and selected by antibiotics in TAP agar medium plates.

### Screening of transgenic alga with the blue light-inducible expression system

**Fig. 3:**
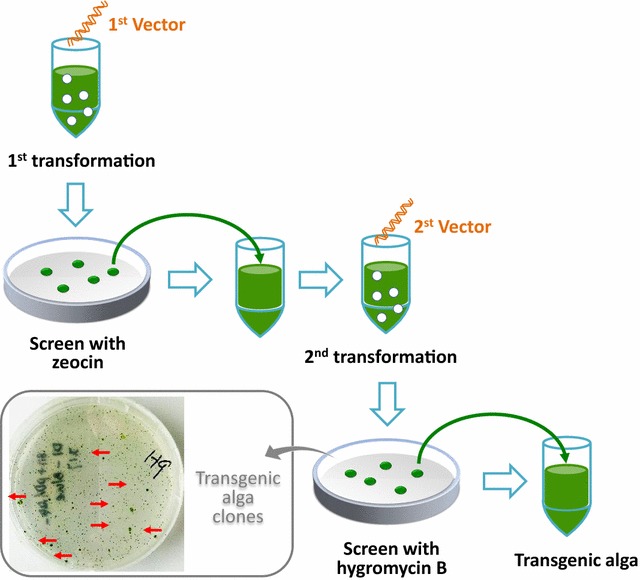
Algal transformation and screening. Step 1: the pDb124-Gal4 BD-CIB1 vector was transformed into CC-849, and the algae were screened with zeocin. Step 2: the pDh124-VP16-CRY2-UAS-amiRNA vector was transformed into transgenic algae from step 1, and the algae were screened with hygromycin B. Red arrows in the picture indicate positive clones, i.e., transgenic algal strains containing the blue light-inducible exogenous gene expression system

Algal cells were transformed and screened through two steps with different antibiotics. First, the cell-wall-deficient *C. reinhardtii* strain was transformed with the pDb124-Gal4 BD-CIB1 vector and screened with zeocin. Second, transgenic algal strains obtained in the first step were transformed with the pDh124-VP16-CRY2-UAS-amiRNA vector and screened with hygromycin B (Fig. 3). Positive clones were purified and verified by sequencing. These results showed that the alga transformation was successful and that the transgenic algal strains contained both vectors required for the blue light-inducible exogenous gene expression system.

### Genomic DNA PCR verification

Transgenic algal strains screened by antibiotics were verified using genomic DNA PCR analysis. Genomic DNA was extracted from CC-849 and transgenic alga using Universal DNA Extraction Kit Ver.3.0 (Takara). PCR was performed with Phanta^®^ Super-Fidelity DNA Polymerase (Vazyme) according to the user manual. All of the amplified products were verified by DNA Sanger sequencing (IGE Biotech., Guangzhou, China).

### Small RNA extraction and reverse transcription

Small RNAs were isolated from CC-849 and transgenic alga using the small RNAiso reagent (Takara) according to the manufacturer’s instructions. Polyadenylation and small RNA reverse transcription were performed using the PrimeScript™ RT Reagent Kit with gDNA Eraser (Perfect Real Time) kit (Takara) according to the user manual.

### Quantitative real-time PCR (qRT-PCR)

To detect the expression levels of miR1166.1 in CC-849 and transgenic alga, qRT-PCR was performed using SYBR Premix Ex Taq™ II (Takara) according to the manufacturer’s instructions and the Applied Biosystems 7300 Real-Time PCR System (Framingham, MA, USA). *U4* snRNA was used as the reference gene for miR1166.1 qRT-PCR detection, and *ACTIN* was used as the reference gene for *psbA* mRNA detection. The primer sequences are listed in Additional file 1: Table S1. Data were processed using the 2^−ΔΔCt^ calculation method, and then were analyzed using *F* test to test the homogeneity of variance and *t* test to determine difference significance.

### Hydrogen detection

CC-849 and transgenic alga (250 ml) were cultured in 500-ml culture bottles sealed with rubber sheet septa until exponential phase in a red light incubator, followed by irradiation with continuous white or blue light to detect hydrogen production. A gas chromatograph was used to detect the concentration of H_2_ (Agilent 7890A; Agilent Technologies Inc., USA). H_2_, O_2_, and N_2_ in the gas samples were separated by a molecular sieve column (type 5 Å; mesh size 60/80; 6 ft. × 1/8 in. × 2.0 mm), and argon was used as the carrier gas. Data were analyzed using *F* test to test the homogeneity of variance, and then using *t* test to determine difference significance.

## Results

### Blue light-inducible exogenous gene expression system

The GAL4 BD domain and VP16 transcription activator were fused with full-length CIB1 and CRY2, respectively. Upon blue light irradiation, CIB1 and CRY2 heterodimerization brought the GAL4 BD and VP16 activator into close proximity, resulting in transcription activation of the gene downstream of the UAS. The absence of blue light reversed the interaction and stopped gene transcription (Fig. 1).

### The effect of blue light on H_2_ photoproduction in transgenic alga

**Fig. 4:**
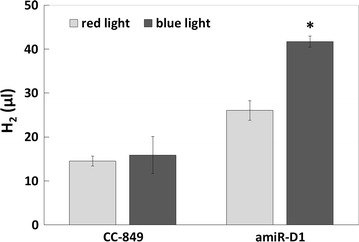
H_2_ production of CC-849 and transgenic alga in continuous red light and after 8-h blue light illumination. 250 ml algal cells were cultured in TAP (Tris–acetate-phosphate) medium at 22 °C, and the *Y*-axis is the total H_2_ yield in the culture bottle

Transgenic alga and CC-849 were cultured in red light until exponential phase and then treated with blue light for 8 h, and the hydrogen production was measured. Hydrogen production was significantly higher in the transgenic alga compared to CC-849 after blue light irradiation (Fig. 4), indicating that blue light induction of amiR-D1 expression affected the hydrogen photoproduction process in *C. reinhardtii*. In the following experiments, we investigated the effects of blue light and white light on target gene transcription activation, and explored the possibility of sustained hydrogen production by repeated induction.

### Blue light irradiation activates artificial miRNA transcription

**Fig. 5:**
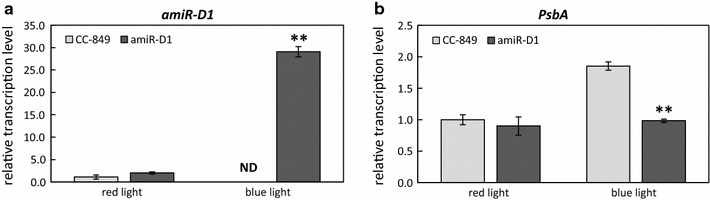
Quantitative transcript analysis of amiR-D1 (**a**) and *psbA* (**b**) in CC-849 and the transgenic algal strain before and after blue light irradiation. *U4* and *ACTIN* were used as the reference genes. *ND* not detected

Since algal cell growth mostly relies on energy from photosynthesis, complete darkness would compromise the algal growth rate. We therefore used red light instead of darkness to reverse CRY2/CIB1 interaction (Additional file 1: Figure S1). The algae were cultured in a red light incubator until exponential phase and then placed under blue light. The transcript levels of amiR-D1 in CC-849 and the transgenic algal strain were analyzed by quantitative PCR before and after blue light irradiation. amiR-D1 transcripts in CC-849 were not detected or very low (due to unspecific binding), while in the transgenic alga, amiR-D1 was up-regulated by more than 14-fold after blue light treatment (Fig. 5a). After blue light irradiation, the *psbA* gene encoding D1 was up-regulated in CC-849 and almost unchanged in amiR-D1 transgenic alga (Fig. 5b). The results demonstrated the successful application of our blue light-inducible expression system in *C. reinhardtii* and the activation of amiR-D1 expression.

### Blue light induces hydrogen production by optogenetically regulating amiR-D1

**Fig. 6:**
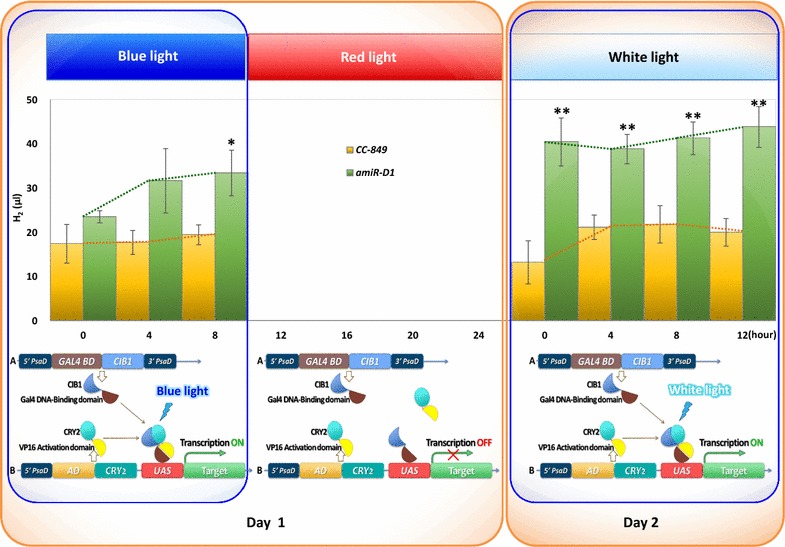
H_2_ production of CC-849 and transgenic alga in blue light, red light, and white light. The column chart shows the total H_2_ yields of equal culture volumes of CC-849 and amiR-D1 transgenic algae in the culture bottles; yield was calculated from the H_2_ peak areas detected by gas chromatography (GC). The diagrams beneath the column chart summarize the blue light-induced transcription mechanism

Hydrogen production of CC-849 and amiR-D1 transgenic alga in red light, blue light, and white light conditions were measured by gas chromatography (GC). The algae were cultured in red light until exponential phase, irradiated with blue light for 8 h, and then returned to red light before the white light irradiation. The first 4 h of blue light irradiation activated hydrogen production significantly in amiR-D1 transgenic alga but not in CC-849. The subsequent 4-h blue light treatment and the white light treatment on the second day only enhanced hydrogen production very slightly. Neither blue light nor white light irradiation showed any effect on CC-849 (Fig. 6). The increased hydrogen production clearly demonstrated that blue light enhanced hydrogen production through optogenetic activation of amiR-D1.

## Discussion

Light-inducible systems have been used in diverse organisms, such as bacteria [17], cyanobacteria [18, 19], yeast [20], and mammalian cells [21, 22], but not in plants including green algae. This may reflect the fact that light is essential to photosynthetic organisms, and light deprivation has detrimental effects on many physiological processes. One exception is cyanobacteria, which are photosynthetic and prokaryotic microorganisms. However, the green/red light-regulated gene expression system used in cyanobacteria relies on cyanobacteriochromes (CBCRs), photoreceptors unique to these organisms [18, 19]. Although CBCRs are promising, they have not yet been shown to be applicable in eukaryotic cells. For this reason, we chose the CRY2/CIB1 system, which has already been successfully applied in many species without requiring any cofactors. To resolve the contradiction of using a light-induction system in a photosynthetic organism, algae were cultured in TAP medium under red light before blue light induction. Unlike darkness, red light can support algal cell photosynthesis. Additionally, the red light was produced using a red LED, which radiates light in a very narrow wavelength range and therefore cannot activate exogenous gene expression. TAP medium contains acetic acid, which provides algal cells energy as the carbon source. These two measures ensured the approximately normal growth of algal cells (Additional file 1: Figure S1B, C).

Full-spectrum white light has been reported as a light source for the CRY2/CIB1 system because no light wavelength has been found to reverse CRY2/CIB1 dimerization [20]. We used both blue light and white light to induce exogenous gene transcription, and both exhibited significant activation effects. Continuous light exposure causes cumulative stress in cultured mammalian cells [24], but we supposed that green algae cells may have better blue light tolerance because it is a photosynthetic organism. The results showed that both blue light and white light could induce hydrogen production, and they did not exhibit different effects. Thus, this blue light-inducible system can be activated by natural light, which is advantageous for industrialized cultivation because natural light is much more convenient and economical.

The blue light-inducible system altogether expressed three exogenous genes: CRY2-VP16, CIB1-GAL4 BD, and the target gene. To make the system easier to use, the three DNA fragments were arranged into two vectors in light of previous reports that a binary GAL4-VP16–UAS transactivation system was successfully applied in the higher plant *Oryza sativa* L. [25, 26]. Accordingly, we tandemly connected CRY2-VP16, UAS, and the target gene together in pDh124, with the following advantages: firstly, transformation is done twice instead of three times; secondly, only two antibiotics have to be added into the medium.

Sulfur deprivation can lead to sustained hydrogen production in *Chlamydomonas*, since the absence of sulfur inhibits photosynthetic activity and results in anaerobic environment in algal cells. Then hydrogenase is released from oxygen inhibition to produce hydrogen. Biochemical characterization of sulfur-deprived alga found that D1 protein is declined significantly. D1 is the reaction-center protein in the PSII complex, and is encoded by *psbA* gene. Accordingly, we chose D1 as the target and designed artificial miRNA to regulate hydrogen production [27, 28].

Hydrogen production in green algae requires electrons from the photosynthetic electron transfer chain to reduce H^+^, so we integrated a recovery time between the activating light irradiation treatments. During the recovery period, algal cells were cultured in red light for 12 h without amiR-D1 expression. We hypothesized that algal cells could produce electrons required for hydrogen production under white light irradiation on the second day, resulting in sustainable hydrogen production. However, the results showed that only the first blue light irradiation induced a significant increase in hydrogen yield, and the second round of white light irradiation did not have an ideal effect. One possible explanation is that cell growth and division had exhausted the available nutrition in the medium, and the cells in a poor growth state could not generate enough electrons. This situation may be improved by continuous culture with sustained fresh substrate feeding in industrialized cultivation.

The blue light-inducible exogenous gene expression system is highly versatile because all of the components in the system can be replaced. The target gene can be conveniently cut and replaced via restriction enzyme cleavage or homologous recombination to express protein coding genes, natural or artificial miRNAs, RNAi sequences, etc. Applicable DNA-binding components include GAL4 BD, zinc-finger proteins (ZFPs), transcription-activator-like effectors (TALEs), the CRISPR–Cas system, and the catalytically inactive dCas9. Possible light sensors include CRY2/CIB1, Phy/PIF, FKBP-FRB, and Magnets. The functional components can have various effects, including recombination (using endonucleases), transcription activation (using transcription activators such as VP16), transcription repression (using transcription repressors or DNA-binding proteins that block the target gene locus), or epigenetic modification (using enzymes such as histone modifiers) [21, 22, 29]. This highly versatile blue light-inducible system has potential as a powerful genome programming tool to control gene expression in green algae and possibly in higher plants, with the effects easily reversed by removing the blue light.

## Conclusions

Previous studies have used nutrient-starved medium or heat treatment to improve hydrogen production by green algae; however, both methods affect algal cell growth and reduce the viability of large-scale algae culture. In this study, we described a blue light-inducible system in *C. reinhardtii* that achieved optogenetic regulation of an artificial miRNA and improved hydrogen production. This system activated amiRNA transcription and consequently suppressed target gene expression in blue light, and the activation was reversed without blue light. Transgenic alga were grown in red light to improve biomass accumulation and transferred to blue or white light to express the amiRNA targeting D1 protein and thus enhance hydrogen production. The hydrogen yield of transgenic alga with the light-inducible system was double that of the control group.

## Additional file

**Additional file 1: Table S1.** Primers used in the experiments. **Figure S1**. Red light condition for algal cells growth. (A) The wavelength distribution of custom-built LED red light incubator with continuous illumination. (B) Photographs of algae at exponential phase in white/red light (the 3rd day). (C) Concentrations of algal cells at exponential phase in white/red light. The algal cell densities are corresponding to the photographs in (B) one by one.
