# Supplementary material for: Optogenetic regulation of artificial microRNA improves H2 production in green alga Chlamydomonas reinhardtii
Source: Biotechnol Biofuels. 2017 Nov 7;10:257. doi: 10.1186/s13068-017-0941-7 (PMC5678773; doi:10.1186/s13068-017-0941-7)
Supplement: Supplementary file 1 — Additional file 1: Table S1. Primers used in the experiments. Figure S1. Red light condition for algal cells growth. (A) The wavelength distribution of custom-built LED red light incubator with continuous illumination. (B) Photographs of algae at exponential phase in white/red light (the 3rd day). (C) Concentrations of algal cells at exponential phase in white/red light. The algal cell densities are corresponding to the photographs in (B) one by one. [file 13068_2017_941_MOESM1_ESM.docx]

**Table S1** Primers used in the experiments.

| **name** | **sequence** | **annotation** |
| --- | --- | --- |
| **PsbA-F** | CCACATGTTAGGTGTTGCTGGTG | Forward primer for qPCR |
| **PsbA-R** | GAAGCGTATTGGAAGATTAGACGACC | Reverse primer for qPCR |
| **ACTIN-F** | ACCCCGTGCTGCTGACTG | Forward primer for qPCR |
| **ACTIN-R** | ACGTTGAAGGTCTCGAACA | Reverse primer for qPCR |
| **miR-D1-RT** | GGTCGTATGCAAAGCAGGGTCCGAGGTATCCATCGCACGCATCGCACTGCATACGACCgcgtat | Reverse transcription primer |
| **U4-RT** | GGTCGTATGCAAAGCAGGGTCCGAGGTATCCATCGCACGCATCGCACTGCATACGACCgagggc | Reverse transcription primer |
| **miR-D1-F** | TTAATGAACCACCGA | Forward primer for qPCR |
| **U4-F** | ATTTCTGTCGGGCCTTTTG | Forward primer for qPCR |
| **Universal R** | GAGCAGGGTCCGAGGT | Universal reverse primer for qPCR |

Figure S1 Red light condition for algal cells growth. (A) The wavelength distribution of custom-built LED red light incubator with continuous illumination. (B) Photographs of algae at exponential phase in white/red light （the 3^th^ day）. (C) Concentrations of algal cells at exponential phase in white/red light. The algal cell densities are corresponding to the photographs in (B) one by one.
